# Generation and Characterization of ORF55/ORF57-Deleted Recombinant *Cyprinid herpesvirus* 2 Mutants with Chimeric Capsid Protein Gene of Grouper Nervous Necrosis Virus

**DOI:** 10.3390/vaccines12010043

**Published:** 2023-12-30

**Authors:** Zizhao Feng, Wenjie Cheng, Mingyang Ma, Chenwei Yu, Ye Zhang, Liqun Lu, Hao Wang, Lang Gui, Dan Xu, Chuanfu Dong

**Affiliations:** 1 National Pathogen Collection Center for Aquatic Animals, Shanghai Ocean University, Shanghai 201306, China; 18022911927@163.com (Z.F.); cwj710574763@163.com (W.C.); 2111202@st.shou.edu.cn (M.M.); m230150423@st.shou.edu.cn (C.Y.); yzhang@shou.edu.cn (Y.Z.); lqlv@shou.edu.cn (L.L.); h-wang@shou.edu.cn (H.W.); lgui@shou.edu.cn (L.G.); 2 National Demonstration Center for Experimental Fisheries Science Education, Shanghai Ocean University, Shanghai 201306, China; 3 Key Laboratory of Freshwater Aquatic Genetic Resources, Ministry of Agriculture, Shanghai Ocean University, Shanghai 201306, China; 4 State Key Laboratory of Biocontrol, School of Life Sciences, Sun Yat-sen University, Guangzhou 510275, China

**Keywords:** CyHV-2, RGNNV, virulence, homologous recombination, viral vector

## Abstract

*Cyprinid herpesvirus* 2 (CyHV-2) is a pathogen that causes significant losses to the global aquaculture industry due to mass mortality in crucian carp and goldfish. This study demonstrates that the ORF55/ORF57 deletion mutants CyHV-2-Δ55-CP and CyHV-2-Δ57-CP obtained through homologous recombination replicate effectively within the caudal fin of *Carassius auratus gibelio* (GiCF) cells and exhibit morphologies similar to the CyHV-2 wild-type strain. Both mutants demonstrated a decrease in virulence, with CyHV-2-Δ57-CP exhibiting a more significant reduction. This serves as a reference for the subsequent development of recombinant attenuated vaccines against CyHV-2. Additionally, both mutants expressed the inserted RGNNV-CP (capsid protein of *Redspotted grouper nervous necrosis virus*) fusion protein gene, and inoculation with CyHV-2-Δ57-CP-infected GiCF cell lysates elicited an antibody response in the grouper. These results indicate that, while ORF55 and ORF57 genes of CyHV-2 are not required for viral replication in vitro, they do play a role in virulence in vivo. Additionally, expression of foreign protein in CyHV-2 suggests that the fully attenuated mutant of CyHV-2 could potentially function as a viral vector for developing subunit vaccines or multivalent recombinant attenuated vaccines.

## 1. Introduction

*Cyprinid herpesvirus* 2 (CyHV-2), also referred to as *Cyvirus cyprinidallo* 2 and herpesviral hematopoietic necrosis virus (HVHNV), is a virus with an icosahedral capsid containing a dsDNA genome and is surrounded by a lipid envelope containing viral glycoproteins. CyHV-2 causes acute mass mortality (up to 100%) in populations of crucian carp (*Carassius auratus*) and its variants such as goldfish (*C. auratus* L.) and gibel carp (*C. auratus gibelio*), resulting in significant economic losses in the aquaculture industry [1]. For environmentally friendly aquaculture, vaccination strategies have demonstrated high effectiveness and cost-effectiveness in protecting fish against various viruses [2]. Previous studies have reported the development of vaccines for CyHV-2 in various forms, including conventional live attenuated [3,4], inactivated [5,6,7,8], DNA [9,10], subunit [11], and live vector vaccines [12,13,14,15,16,17]. However, currently, there is no commercially available licensed vaccine against CyHV-2. Additionally, immersion and oral vaccines are better suited for large-scale operations in fish farms when immunizing relatively affordable juvenile carp and goldfish [18]. Live attenuated vaccines, on the other hand, tend to be highly immunogenic and closely resemble natural pathogen infections due to their ability to replicate within the host and stimulate robust cellular responses related to both innate and adaptive immune systems [19]. Hence, attenuated live vaccines can elicit long-lasting immunity within the host through oral or immersion routes. However, a major concern with conventional live attenuated vaccines is the risk of reversion to virulence [20]. Recombinant attenuated vaccines, involving the deletion of virulence-related genes, are a type of live vaccine that cannot be easily reversed under natural conditions. Furthermore, recombinant attenuated vaccines represent a crucial avenue for the advancement of aquaculture vaccines, given their high stability, potent immunogenicity, and guaranteed safety [21]. To date, no studies have reported the creation of recombinant attenuated vaccines against CyHV-2. 

CyHV-2 is a member of the genus *Cyvirus* in the family Alloherpesviridae, to which *Cyvirus cyprinidallo* 1 (*Cyprinid herpesvirus* 1, CyHV-1; carp pox virus), *Cyvirus cyprinidallo* 3 (*Cyprinid herpesvirus* 3, CyHV-3; koi herpesvirus, KHV), and *Cyvirus anguillidallo* 1 (*Anguillid herpesvirus* 1, AngHV-1) also belong [22]. Phylogenetically, all three strains of cyprinid herpesviruses (CyHVs) are closely related, with CyHV-2 and CyHV-3 being slightly more closely related to one another than to CyHV-1 [23]. In 2018, a patent was granted in the United States for a recombinant attenuated CyHV-3 vaccine with mutations in ORF56 and ORF57 genes [21]. This vaccine has demonstrated exceptional immunoprotective potential against CyHV-3 in both common carp and koi [24]. Indeed, subsequent research has identified the ORF57 gene as the essential virulence factor in the double deletion of ORF56 and ORF57 genes [25]. However, the function of the ORF57 protein in CyHV-2 and CyHV-3 remains to be determined. The identification of ORF57 protein as a critical virulence factor of CyHV-3 has provided a crucial target for further development of recombinant attenuated vaccines against CyHV-2 [26]. Additionally, homology analysis and RNA interference (RNAi) experiments were performed to investigate the ORF57 and thymidine kinase (TK) genes of CyHVs [27]. The findings suggest that the ORF57 and TK genes are conserved in CyHVs and may have an impact on the virulence of CyHV-2. Furthermore, the TK gene was revealed to be unnecessary for replication in vitro but pertinent to virulence in vivo as shown in numerous herpesviruses including CyHV-3 [28,29,30,31]. Therefore, this study selected the ORF55 (TK) and ORF57 genes as targets for constructing CyHV-2 recombinant mutants. 

The *Redspotted grouper nervous necrosis virus* (RGNNV) is a non-enveloped, small icosahedral virus (25–30 nm) whose genome contains two positive-sense, single-stranded RNA molecules: RNA1 and RNA2 [32]. RGNNV belongs to the genus *Betanodavirus* in the family Nodaviridae, along with *Barfin flounder nervous necrosis virus* (BFNNV), *Striped jack nervous necrosis virus* (SJNNV), and *Tiger puffer nervous necrosis virus* (TPNNV) [22]. Viral nervous necrosis (VNN) disease caused by *Betanodavirus*, also known as viral encephalopathy and retinopathy (VER), and viral encephalopathy, is a highly destructive disease that negatively impacts at least 57 species of marine fish and 13 species of freshwater fish (including goldfish) worldwide, resulting in financial losses to the aquaculture industry [33]. The only structural protein of *Betanodavirus*, capsid protein (CP), encoded by RNA2, is a promising candidate for future vaccine development because of its ability to elicit effective immune responses [33]. A recently reported, recombinant bivalent live viral vectored vaccine candidate expresses the major protective antigen domain of NNV-CP in attenuated *Viral hemorrhagic septicemia virus* (VHSV) and has been shown to protect against lethal VHSV and NNV challenge [34].

Viral vector vaccines, derived from non-pathogenic virions whose genomes have been modified by inserting one or more genes encoding for the heterologous antigens, can express several heterologous antigens to elicit strong immune responses and increase cellular immunity in hosts [35]. These types of vaccines have been widely utilized in both human and veterinary medicine [36,37]. Among these, herpesviruses have become significant vectors because of their ability to carry large exogenous genes, infect only a limited range of hosts, express envelope glycoproteins on, and elicit both cellular and humoral immune responses [36,38]. However, only a handful of viruses, including baculovirus [14,15], adenovirus [39,40], *Semliki Forest virus* (SFV) [41], *Salmon pancreas disease virus* (Salmonid alphavirus, SAV) [42,43], *Viral hemorrhagic septicemia virus* (VHSV) [34], *Infectious hematopoietic necrosis virus* (IHNV) [44], and *Ictalurid herpesvirus 1* (IcHV-1; channel catfish herpesvirus, CCV) [45], have been utilized as viral vectors for the creation of aquaculture vaccines or expression systems. In this study, we inserted the RGNNV-CP gene into the genome of CyHV-2 and conducted an initial assessment of its potential as a viral vector for expressing heterologous proteins.

## 2. Materials and Methods

### 2.1. Animals, Cells and Virus

Gibel carps (*C. auratus gibelio*) var. CAS V weighing 8 ± 2 g and 200 ± 20 g, gibel carps (*C. auratus gibelio*) var. CAS III weighing 200 ± 20 g, Fang Zheng crucian carps (*C. auratus gibelio*) weighing 200 ± 20 g, and white crucian carps (*C. auratus cuvieri*) weighing 200 ± 20 g were obtained from a fishery located in the Nanhai District of Foshan, Guangdong Province, China. Additionally, goldfish (*C. auratus* L.) weighing 8 ± 2 g (one year old) and 100 ± 10 g (over two years old) were obtained from a fishery in Dianshan Lake Town, Kunshan, Jiangsu Province, China. All of the fish mentioned above, confirmed as CyHV-2-negative through PCR testing, were maintained in recirculating aquaculture systems at a temperature of 25 °C until the start of the experiments.

Orange-spotted groupers (*Epinephelus coioides*) weighing 200 ± 20 g were acquired from a fishery in the Hailing District of Yangjiang, Guangdong Province, China. The Groupers were confirmed to be NNV-negative by PCR testing and housed in a seawater recirculating aquaculture system at a temperature of 28 °C until the experiments.

The *C. auratus gibelio* caudal fin (GiCF) cell line, established and maintained in our laboratory [46], was cultured in medium 199 (Gibco, Grand Island, NY, USA) supplemented with 10% fetal bovine serum (Gibco, NY, USA) at 27 °C.

The CyHV-2 wild-type (WT) strain YC01 (Genebank: MN593216.1), isolated from diseased gibel carp in our previous study [47], was propagated in GiCF cells (MOI = 1). The infected GiCF cell lysate was harvested at 5 days after infection (dpi) and stored at −80 °C.

The virus-like particles of NNV (NNV VLP) were generously provided by Dr. Junfeng Xie, State Key Laboratory of Biocontrol/School of Life Sciences, Sun Yat-sen University, Guangzhou, Guangdong, China.

### 2.2. Construction of Transfer Vectors and Virus Recombinants

The pUC18 vector was employed for the construction of recombinant transfer vectors, namely pUC18-Δ55-CP and pUC18-Δ57-CP. These vectors were designed to incorporate the CMV promoter, the full-length RGNNV capsid protein gene (Genebank: AF534998.3), puromycin resistance gene (Puro^r^), enhanced green fluorescent protein (EGFP) gene, as well as 1 kb upstream and downstream arms of the ORF55 or ORF57 gene. In brief, the corresponding segments were initially obtained using the primers listed in Table 1 and KOD DNA Polymerase (TOYOBO, Osaka, Japan). Subsequently, the CP-Puro fusion protein segment was generated through overlap extension PCR, along with the 55UD and 57UD segments which contain double restriction enzyme sites between the upstream and downstream arms. Using the ClonExpress^®^ II One Step Cloning Kit (Vazyme, Nanjing, China), the CP-Puro fusion protein segment was ligated into the *Eco*RI/*Bam*HI-digested plasmid vector pEGFP-N3 to obtain pEGFP-N3-CP-Puro^r^. Additionally, the 55UD and 57UD segments were separately ligated into the *Bam*HI/*Eco*RI-digested plasmid vector pUC-18, resulting in pUC-18-55UD and pUC-18-57UD. Finally, the NNV-CP fusion protein expression cassette was obtained from pEGFP-N3-CP-Puro^r^ and subsequently ligated into the *Kpn*I/*Eco*RI-digested plasmid vectors pUC-18-55UD and the *Bam*HI/*Eco*RI-digested plasmid vectors pUC-18-57UD to obtain the recombinant transfer vectors, pUC18-Δ55-CP and pUC18-Δ57-CP, respectively.

GiCF cells were individually transfected with the recombinant transfer vectors pUC18-Δ55-CP and pUC18-Δ57-CP using the EZ 3000 Plus transfection reagent (ELGBIO, Guangzhou, China) and maintained in 10% FBS medium 199 at 27 °C for 24 h. Subsequently, GiCF cells were infected with the CyHV-2-WT strain (MOI = 1) and kept in 2% FBS medium 199 at 27 °C, to generate the CyHV-2 recombinant mutants by homologous recombination.

Two rounds of puromycin selection (Sigma-Aldrich, St. Louis, MO, USA) were performed using a final concentration of 1 μg/mL to enrich the CyHV-2 recombinant mutants by eliminating CyHV-2-WT-infected cells lacking puromycin resistance. Subsequently, recombinant mutants CyHV-2-Δ55-CP and CyHV-2-Δ57-CP were purified to homogeneity by multiple rounds of fluorescent plaque purification. In brief, green fluorescent cell foci were picked by aspiration, and subsequently used for infecting new cells through the limited dilution method, repeating this process iteratively to obtain homogeneous recombinant strains. The purity of obtained CyHV-2 recombinant mutants was confirmed via the nested-PCR amplification technique along with sequencing analysis.

### 2.3. DNA Extraction and Sequence Analysis

Genomic DNA was extracted using the FastPure Cell/Tissue DNA Isolation Mini Kit (Vazyme, Nanjing, China). Whole-genomic sequences of CyHV-2-WT, CyHV-2-55-CP, and CyHV-2-57-CP strains were sequenced utilizing the Illumina NovaSeq PE150 platform provided by Beijing Novogene Bioinformatics Technology Co., Ltd. (Beijing, China). Trinity software (version 2.14.0) was used to assemble whole-genomic sequences against reference sequence YC01 (GenBank: MN593216.1) if CyHV-2 wild-type strain, and unmapped reads were retrieved employing Bowtie2 tool (version 2.4.5). Using the assembled sequence of CyHV-2-WT as the reference, the final assembly results were aligned by DNAMAN (version 9.0.1) and SnapGene (version 2.3.2) software.

### 2.4. Western Blotting Analysis

The expression of the NNV-CP fusion protein derived from the CyHV-2 recombinant mutants within GiCF cells was assessed using Western blotting (WB). Briefly, GiCF cells were collected at 48 h post-infection with CyHV-2-WT, CyHV-2-Δ55-CP, or CyHV-2-Δ57-CP (MOI = 1) and lysed using IP lysis buffer (Pierce, Rockford, IL, USA). Total proteins in the lysates were separated by SDS polyacrylamide gel electrophoresis, transferred to PVDF (polyvinylidene fluoride) membranes (Merck, Boston, MA, USA), and blocked with 5% fat-free milk in PBS-T (0.05% Tween 20). The membranes were then incubated with primary antibody (1:2000 dilution), including rabbit anti-NNV VLP polyclonal antibody (provided by Dr. Junfeng Xie) or mouse anti-GFP monoclonal antibody (Abmart, Shanghai, China). After washing the membranes with PBS-T at room temperature, secondary antibodies (1:5000 dilution), HRP-conjugated goat anti-rabbit or anti-mouse IgG (Promega, Madison, WI, USA), were added. The reactive bands were visualized using Tanon High-sig ECL Western Blotting Substrate (Tanon, Shanghai, China).

### 2.5. Indirect Immunofluorescence Analysis

GiCF cells infected with CyHV-2-WT, CyHV-2-Δ55-CP, or CyHV-2-Δ57-CP (MOI = 1) were incubated at 27 °C for 48 h. Subsequently, infected cells were fixed with 4% paraformaldehyde for 15 min and permeabilized with 0.5% Triton X-100 for 20 min followed by blocking with 10% goat serum for 30 min. Incubation with rabbit anti-NNV VLP polyclonal antibody as the primary antibody (1:1000 dilution) and Alexa Fluor 555-conjugated (red fluorescence) goat anti-rabbit IgG (Abcam, Cambridge, UK) as the secondary antibody (1:1000 dilution) was performed. Finally, cell nuclei were stained using DAPI (Abcam, Cambridge, UK). The cell samples were observed under a TCS SP8 confocal microscope (Leica, Wetzlar, Germany).

### 2.6. Virus Purification and Transmission Electron Microscope (TEM)

The infected GiCF cell lysates were prepared as described in Section 2.1, followed by three freeze–thaw cycles. Cell debris was removed by centrifugation at 5000× *g* for 30 min at 4 °C, and the supernatants were further centrifuged at 80,000× *g* for 2 h at 4 °C to pellet virus. The viral pellets were then resuspended in sterile phosphate-buffered saline (PBS, pH 7.4) after being washed with PBS. Subsequently, the resulting suspensions were subjected to centrifugation through discontinuous sucrose gradients (20%, 30%, 40%, 50%, and 66%) at 150,000× *g* for 1 h at 4 °C. Finally, the viral bands were carefully extracted and pelleted by centrifugation in sterile PBS at 150,000× *g* for 2 h at 4 °C. To observe the basic morphological structure of viral particles, the purified viruses were resuspended in PBS and examined using TEM (JEOL JEM-1400 electron microscope, Tokyo, Japan) after negative staining with 3% phosphotungstic acid (pH 7.2–7.4).

To observe the intracellular structure of viral particles, GiCF cells were infected with CyHV-2-WT, CyHV-2-Δ55-CP, or CyHV-2-Δ57-CP (MOI = 1), and infected cells collected at 2 dpi underwent TEM assay following staining with uranyl acetate–lead citrate.

### 2.7. TCID_50_ Assay

The TCID_50_ assay was performed to determine the viral replication kinetics in GiCF cells. GiCF cells were infected with CyHV-2-WT, CyHV-2-Δ55-CP, or CyHV-2-Δ57-CP (MOI = 1) and incubated at 27 °C. The virus-infected cells were collected daily from day one to seven post-infection and lysed by two freeze–thaw cycles. Subsequently, the viral titers of the lysates were analyzed using TCID_50_ assays based on the Reed–Muench method [48].

### 2.8. In Vivo Virulence Assay

Due to the partial attenuation after serial passages in cell cultures, it is necessary to screen suitable susceptible hosts for CyHV-2-WT. Twenty individuals of each commonly cultured crucian carp variety, including gibel carp var. CAS V (8 ± 2 and 200 ± 20 g), gibel carp var. CAS III (200 ± 20 g), Fang Zheng crucian carp (200 ± 20 g), white crucian carp (200 ± 20 g), and goldfish (8 ± 2 and 100 ± 10 g), were separately challenged using both intraperitoneal injection (at a dose of 10^6^ TCID_50_) and immersion (in water with a final virus concentration of 10^3^ TCID_50_/mL for 2 h or 2 days) methods. Subsequently, the fish were maintained in recirculating aquaculture systems at a temperature of 25 °C. To activate latent CyHV-2 infection, temperature stress was applied from the 14th day post-infection by gradually reducing the water temperature from 25 °C to 15 °C at a rate of 1 °C per day followed by an equal rate of temperature increase back to 25 °C. The fish were observed daily for 2 months and the survival rates were recorded for each group.

The virulence assays for CyHV-2-Δ55-CP and CyHV-2-Δ57-CP strains were conducted on goldfish weighing 8 ± 2 g. Goldfish were divided into three groups with 40 fish per group, which were immersed in continuously aerated water with final virus concentrations of 10^2^ TCID_50_/mL, 10^3^ TCID_50_/mL, and 10^4^ TCID_50_/mL, respectively, for 2 days, ensuring comprehensive exposure to the virus. Moreover, the same volume of M199 was used as in the negative control group. Subsequently, the goldfish were maintained in recirculating aquaculture systems at a constant temperature of 25 °C throughout the entire assay period. Goldfish symptoms and survival rates within each group were monitored daily while PCR verification was performed on three randomly selected dead goldfish from each group. In each CyHV-2 strain, these assays were repeated three times.

To assess the in vivo replication capabilities of CyHV-2-Δ55-CP and CyHV-2-Δ57-CP, gibel carps (var. CAS V) weighing 200 ± 20 g were randomly divided into three groups with 70 fish per group and subsequently infected with CyHV-2-WT, CyHV-2-Δ55-CP and CyHV-2-Δ57-CP via intraperitoneal injection at a dose of 4 × 10^6^ TCID_50_, respectively. The water temperature was maintained at 25 °C throughout the entire experiment. At 24 h post-infection and every 2 days thereafter, liver, spleen, and kidney samples were collected from nine randomly selected fish in each group. Tissues from every three fish were homogenized to generate one sample for viral titer determination using qPCR.

### 2.9. Absolute Quantitative PCR (qPCR)

Viral replication in gibel carps was evaluated by selecting the ORF72 gene encoding the major capsid protein of CyHV-2. The copy number of the ORF72 gene was quantified using a quantitative Real-Time PCR assay with primers 72qF (5′-GCGGATACGTTGGACGATCT-3′) and 72qR (5′-CTCGGCTCTGATGGTGTTGT-3′). A standard curve generated through gradient dilution of plasmid pGEX-4T-3-ORF72 was used for normalization purposes. Absolute qPCR was performed using Polarsignal qPCR mix (MIKX, Shenzhen, China) in the Roche LightCycler 480 system (Roche Diagnostics, Basel, Switzerland) under the following conditions: 94 °C for 20 s, followed by 40 cycles consisting of 94 °C for 10 s, 56 °C for 10 s and 72 °C for 10 s. Three independent biological replicates were conducted for each CyHV-2 strain, and three technical replicates were conducted for each qPCR assay.

### 2.10. NNV-Specific IgM Determined via ELISA

There was a more significant decrease in virulence observed in CyHV-2-Δ57-CP, which was selected for the preliminary assessment of its ability to induce antibody response in groupers. Orange-spotted groupers weighing 200 ± 20 g were intraperitoneally injected with 200 μL of infected GiCF cell lysates (10^9^ copies/mL) of CyHV-2-WT, CyHV-2-Δ57-CP, and 0.1% formalin-inactivated GiCF cell lysates of CyHV-2-Δ57-CP, respectively. Serum samples (*n* = 6) were collected on the 21st day post-injection. The levels of anti-NNV antibodies in sera were evaluated using indirect enzyme-linked immunosorbent assay (ELISA). ELISA plates (Nunc Maxisorp, Fisher Scientific, MA, USA) were coated overnight at 4 °C with NNV-VLP in coating buffer (100 mM bicarbonate/carbonate, pH 9.6), followed by blocking with 5% goat serum in PBST for one hour at room temperature. After the washing steps, serum samples diluted at a ratio of 1:10 in PBST were added to the plate and incubated for one hour at room temperature. Subsequently, the plate was incubated with mouse anti-grouper IgM-specific monoclonal antibodies (1:5000 dilution) (provided by Prof. Hui Gong, Biotechnology Institute, Fujian Academy of Agricultural Sciences, Fuzhou, Fujian, China) for one hour at room temperature, which was detected by HRP-conjugated goat anti-mouse IgG (1:5000 dilution) (Promega, WI, USA) following the same procedure. Finally, the color reaction was developed by adding 100 μL per well of a single solution of 3,3′,5,5′-tetramethylbenzidine (Tiangen, Beijing, China) for 20 min and stopped by adding 50 µL of 2 M sulfuric acid. Absorbance (optical density) was measured at a wavelength of 450 nm.

### 2.11. Statistics Analysis

Statistical analysis was carried out using GraphPad Prism 9.5.1. Two-way analysis of variance (ANOVA) was employed to analyze viral replications both in vitro and in vivo. Survival curves of goldfish infected with CyHV-2 were analyzed using the Kaplan–Meier method. ELISA data were analyzed using one-way ANOVA. Results are presented as mean ± standard error of the mean (SEM). Statistical significance was represented as follows: significant differences (* *p* < 0.05), very significant differences (** *p* < 0.01), and highly significant differences (*** *p* < 0.001).

## 3. Results

### 3.1. Construction of the CyHV-2 Recombinant Mutants

**Figure 1:**
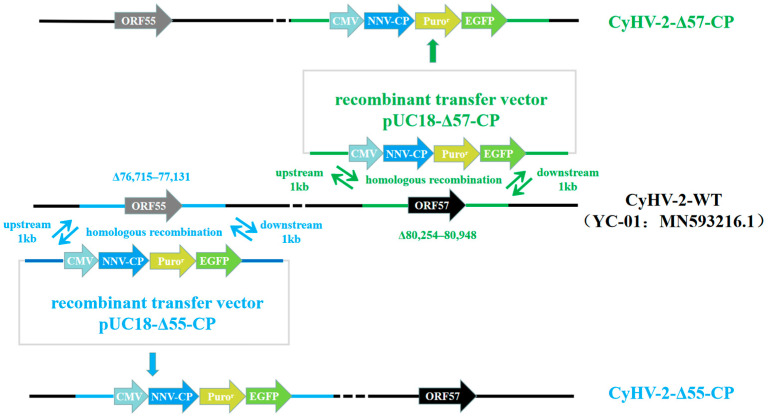
Construction of CyHV-2-Δ55-CP and CyHV-2-Δ57-CP. The ORF55 or ORF57 of the CyHV-2 genome was replaced by homologous recombination with transfer vectors containing the CMV promoter, NNV-CP gene, puromycin resistance gene, and EGFP gene.

**Figure 2:**
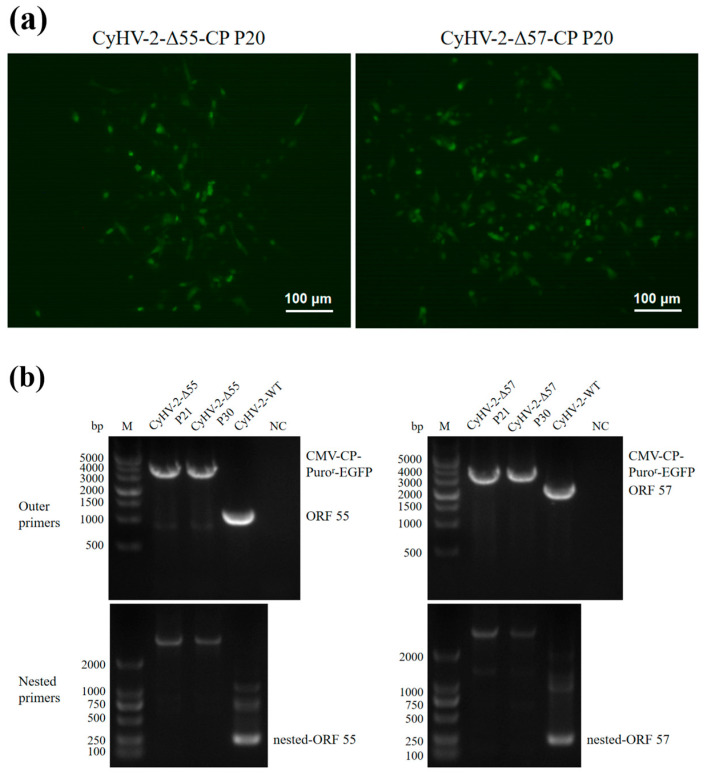
Purity assessment of CyHV-2-Δ55-CP and CyHV-2-Δ57-CP. (**a**) Fluorescence plaques of the CyHV-2 recombinant mutants in GiCF. (**b**) Nested-PCR targeting ORF55 or ORF57. No ORF55 or ORF57 amplicons were detected in both the 21st and 30th passage CyHV-2 recombinant mutants.

**Figure 3:**
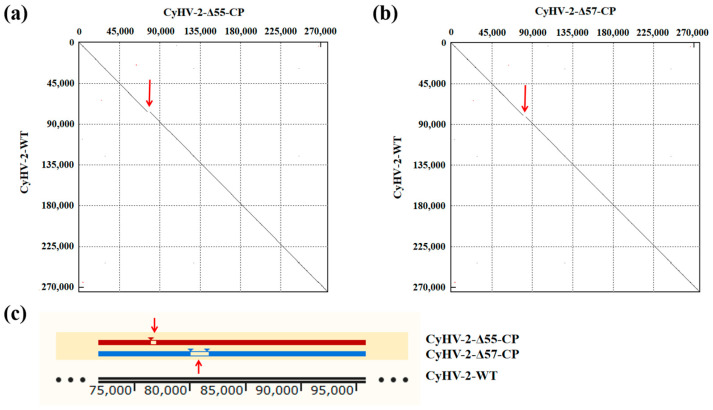
Resequencing of the whole genomes of the CyHV-2 recombinant mutants. (**a**) The dot matrix alignment graph between CyHV-2-Δ55-CP and CyHV-2-WT. (**b**) The dot matrix alignment graph between CyHV-2-Δ57-CP and CyHV-2-WT. (**c**) Resequencing of the whole genomes of the CyHV-2 recombinant mutants. Multiple sequence alignment of CyHV-2-Δ55-CP, CyHV-2-Δ57-CP, and CyHV-2-WT. The gaps indicated by the red arrows in the figure represent the replacement of CyHV-2 ORF55 or ORF57 with the NNV-CP fusion protein expression cassette.

Recombinant transfer vectors pUC18-Δ55-CP and pUC18-Δ57-CP were constructed as shown in Figure 1. GiCF cells were transfected with pUC18-Δ55-CP and pUC18-Δ57-CP, respectively, and then infected with CyHV-2-WT for recombination. Plaques exhibiting green fluorescence activity were selected after two rounds of puromycin selection to enrich recombinant CyHV-2 mutants. These mutants were further purified through eighteen rounds of fluorescent plaque purification (Figure 2a). After 1 and 10 passages (P21 and P30) in GiCF cells, the absence of ORF55 and ORF57 genes was verified in recombinant mutants CyHV-2-Δ55-CP and CyHV-2-Δ57-CP, respectively, using nested-PCR (Figure 2b). Furthermore, the whole-genome sequencing confirmed that only the target gene (ORF55 or ORF57) had been replaced by the NNV-CP fusion protein expression cassette (Figure 3). These results indicate that CyHV-2 recombinant mutants CyHV-2-Δ55-CP and CyHV-2-Δ57-CP have been successfully generated, and their purity meets the requirements for subsequent experiments.

### 3.2. Characteristics of the CyHV-2 Recombinant Mutants

**Figure 4:**
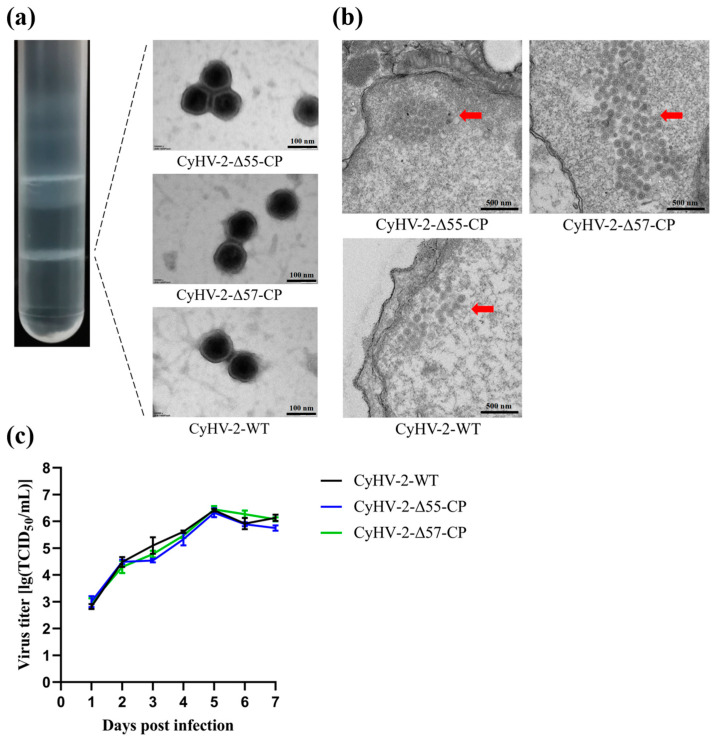
Characteristics of CyHV-2-Δ55-CP and CyHV-2-Δ57-CP. (**a**) Virion images photographed by a transmission electron microscope. Purified recombinant and wild-type CyHV-2 virions treated by negative staining methods. Scale bar = 100 nm. (**b**) Transmission electron micrograph of infected GiCF cells. Red arrows indicate the arrangement of recombinant and wild-type CyHV-2 nucleocapsids in the nucleus. Scale bar = 500 nm. (**c**) Replication kinetics of CyHV-2-Δ55-CP and CyHV-2-Δ57-CP were evaluated in GiCF cells. At the indicated time points, cell samples were collected, and viral titers were determined using TCID_50_ assays.

To analyze the morphology of CyHV-2-Δ55-CP and CyHV-2-Δ57-CP virions, purified virus particles were negatively stained with phosphotungstic acid before being observed under TEM. Imaging examination revealed that both CyHV-2-Δ55-CP and CyHV-2-Δ57-CP virions were enveloped, icosahedral particles, and closely resembled CyHV-2-WT virions (Figure 4a). Transmission electron microscopy (TEM) observations of GiCF cells infected with any one of these CyHV-2 strains revealed a similar and orderly arrangement of numerous capsomers within the cell nucleus (Figure 4b). This suggests that the deletion of either the ORF55 or ORF57 gene from CyHV-2 did not have any discernible influence on viral morphology or structure. Additionally, the replication abilities of CyHV-2-Δ55-CP and CyHV-2-Δ57-CP were evaluated in GiCF cells through in vitro experiments (Figure 4c). Cells were infected with CyHV-2-Δ55-CP, CyHV-2-Δ57-CP, or CyHV-2-WT strains, and viral titers were quantified at the designated time points post-infection. The replication abilities of CyHV-2-Δ55-CP, CyHV-2-Δ57-CP, and CyHV-2-WT in GiCF cells were found to be comparable, as depicted in Figure 4c (*p* > 0.05). Furthermore, all three variants reached their peak titers at 5 dpi with measurements of 6.50, 6.23, and 6.41 TCID_50_/mL, respectively. These results suggest that the replacement of the ORF55 or ORF57 gene with an NNV-CP fusion protein expression cassette does not have any discernible impact on the replication ability of CyHV-2 in GiCF cells.

### 3.3. The Expression of the NNV-CP Fusion Protein

**Figure 5:**
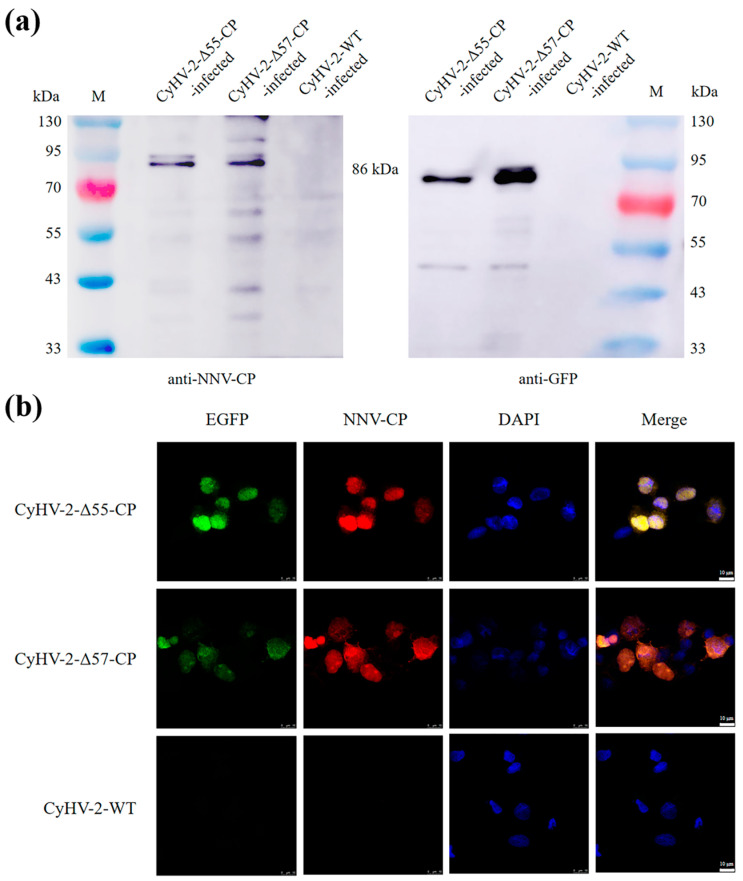
Validation of the NNV-CP fusion protein expression in CyHV-2-Δ55-CP and CyHV-2-Δ57-CP by Western blotting (**a**) and indirect immunofluorescence assay (**b**). (**a**) At 48 h post-infection, the NNV-CP fusion protein was detected with the rabbit polyclonal antibody against NNV VLP and the mouse monoclonal antibody against GFP, respectively. (**b**) At 48 h post-infection, the NNV-CP fusion protein was detected using the rabbit polyclonal antibody against NNV VLP, followed by Alexa Fluor 555-conjugated goat anti-rabbit IgG (red). Cell nuclei were stained using DAPI (blue). Scale bar = 10 μm.

The expression of the NNV-CP fusion protein by CyHV-2-Δ55-CP and CyHV-2-Δ57-CP was evaluated in GiCF cells. At 2 dpi, Western blotting assays confirmed the presence of the expected 86 kDa NNV-CP fusion protein in infected GiCF cell lysates of both CyHV-2 recombinant mutants (Figure 5a). Furthermore, indirect immunofluorescence assays conducted on fixed cells at 2 dpi revealed co-localization between enhanced green fluorescent protein (EGFP) and the rabbit polyclonal antibody against NNV VLP, confirming expression of the NNV-CP fusion protein within GiCF cells. Fluorescence signals were observed in both the cytoplasm and the nucleus (Figure 5b), suggesting that the NNV-CP fusion protein still maintains the nuclear localization function of NNV-CP [49].

### 3.4. Virulence Attenuation in the CyHV-2 Recombinant Mutants

**Figure 6:**
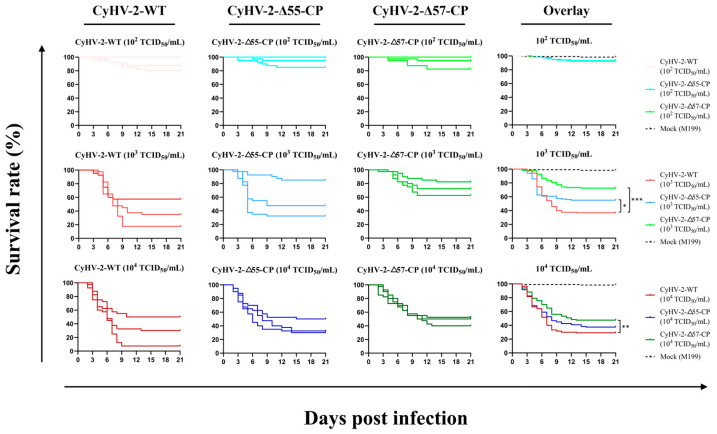
The virulence of recombinant and wild-type CyHV-2 strains in goldfish. On day 0, goldfish were infected for 2 days by immersion in water with virus concentrations of 10^2^ TCID_50_/mL, 10^3^ TCID_50_/mL, and 10^4^ TCID_50_/mL, respectively. The nine panels at left show the survival curves observed for three replicates. The three overlay panels at right show the cumulative survival curves based on the three replicates. Statistical significance was represented as follows: * *p* < 0.05, ** *p* < 0.01, and *** *p* < 0.001.

To identify suitable animal models for further experiments, a challenge experiment using CyHV-2-WT was conducted on various common crucian carp varieties as shown in Table 2. Results showed that only juvenile goldfish weighing 8 ± 2 g (one year old) exhibited mortality, possibly due to other varieties’ stronger disease resistance resulting from selective breeding. A recent study indicates that, although CyHV-2 showed a higher susceptibility in adult goldfish, it was more permissive to replication in larvae, resulting in rapid systemic infection and high mortality in juvenile goldfish [50]. Moreover, prolonged immersion in virus-contaminated water is more likely to induce acute mortality in goldfish. Therefore, challenge experiments were performed using juvenile goldfish to evaluate the virulence of CyHV-2-Δ55-CP and CyHV-2-Δ57-CP (Figure 6). Fish infected with either mutant or wild-type strain displayed typical clinical symptoms such as hemorrhaging, anorexia, lethargy, and bottom-dwelling behavior. The mean survival rates for each group are presented in Table 3.

In the challenge experiment with a virus concentration of 10^2^ TCID_50_/mL, all CyHV-2 strains only induced mortality in a few weak individuals without any significant statistical differences observed among the groups (*p* > 0.05). However, in the challenge experiment with a virus concentration of 10^3^ TCID_50_/mL, compared to the CyHV-2-WT-challenged group, both the CyHV-2-Δ55-CP- and CyHV-2-Δ57-CP-challenged groups showed higher survival rates with significant (* *p* < 0.05) and highly significant (*** *p* < 0.001) differences, respectively. Moreover, in the challenge experiment with a virus concentration of 10^4^ TCID_50_/mL, only the CyHV-2-Δ57-CP-challenged group demonstrated a very significant difference in survival rate compared to the CyHV-2-WT-challenged group (** *p* < 0.01).

**Figure 7:**
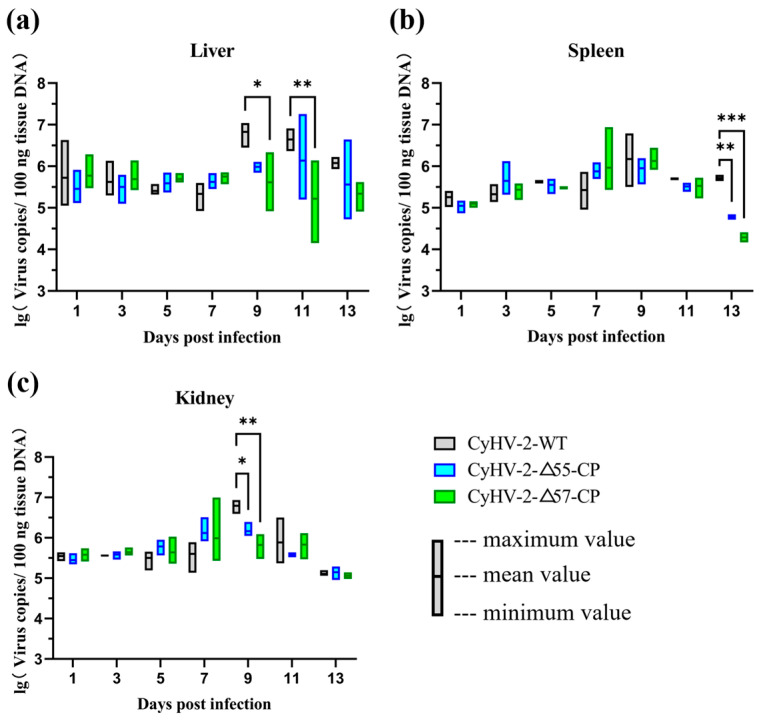
The replication of recombinant or wild-type CyHV-2 strains in gibel carp. On day 0, gibel carps were infected by intraperitoneal injection at a viral dose of 4 × 10^6^ TCID_50_. Liver (**a**), spleen (**b**) and kidney (**c**) tissues were collected for absolute qPCR targeting CyHV-2 ORF72. Statistical significance was represented as follows: * *p* < 0.05, ** *p* < 0.01, and *** *p* < 0.001.

Furthermore, in vivo replication capabilities of CyHV-2-Δ55-CP and CyHV-2-Δ57-CP have been evaluated in gibel carps (var. CAS V). As shown in Figure 7, the copy numbers of CyHV-2-WT in liver, spleen, and kidney reached their peak at 9 days post-infection, while the CyHV-2 recombinant mutants, although exhibiting higher copies in some samples, remained relatively stable overall and were gradually reduced. In the liver, compared to the CyHV-2-WT-challenged group, only the copy numbers in the CyHV-2-Δ57-CP-challenged group showed significant (* *p* < 0.05) and very significant (** *p* < 0.01) differences at 9 and 11 days post-infection, respectively (Figure 7a). In the spleen, copy numbers of CyHV-2-Δ55-CP and CyHV-2-Δ57-CP demonstrated very significant (** *p* < 0.01) and highly significant (*** *p* < 0.001) differences at 13 dpi, respectively, compared with CyHV-2-WT (Figure 7b). Similarly, in the kidney, copy numbers of CyHV-2-Δ55-CP and CyHV-2-Δ57-CP showed significant (* *p* < 0.05) and very significant (** *p* < 0.01) differences at 9 dpi, respectively, compared with CyHV-2-WT (Figure 7c).

In conclusion, these results suggest that both CyHV-2-Δ55-CP and CyHV-2-Δ57-CP showed partial attenuation of virulence which was more obvious in CyHV-2-Δ57-CP.

### 3.5. Infected GiCF Cell Lysates of CyHV-2-Δ57-CP Induced Antibody Response in Grouper

**Figure 8:**
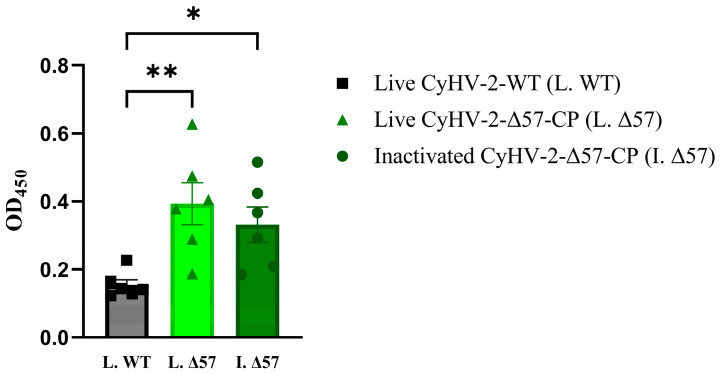
NNV-specific IgM determined by ELISA. Twenty-one days post-vaccination, serum samples were collected from vaccinated grouper, and the levels of NNV-specific IgM were determined by ELSA. Statistical significance was represented as follows: * *p* < 0.05 and ** *p* < 0.01.

On the 21st day post-vaccination, blood samples were collected from six groupers in each group for subsequent anti-NNV specific IgM level determination using indirect ELISA assay. The results revealed that the presence of specific anti-NNV IgM antibodies in the serum of groupers injected with either cell culture suspension of CyHV-2-Δ57-CP or 0.1% formalin-inactivated suspension of CyHV-2-Δ57-CP (Figure 8). Moreover, compared to the control group injected with cell culture suspension of CyHV-2-WT, injection of cell culture suspension of CyHV-2-Δ57-CP significantly induced a production of specific anti-NNV IgM antibodies with very significant differences (** *p* < 0.01), while injection of 0.1% formalin-inactivated suspension of CyHV-2-Δ57-CP only resulted in significant differences (* *p* < 0.05). These results suggested that live CyHV-2-Δ57-CP suspension may elicit higher titers of anti-NNV IgM antibodies compared to formalin-inactivated suspension.

## 4. Discussion

The global crucian carp and goldfish industries have suffered significant economic losses due to the epidemic of herpesviral hematopoietic necrosis (HVHN) disease [1]. Therefore, there is an urgent need for an effective vaccine that allows mass immunization of cost-effective juveniles, such as crucian carp fry. Attenuated vaccines have shown the most promising overall performance for CyHV-3 vaccines so far [26]. Identifying key virulence genes that are non-essential for in vitro amplification is crucial for developing recombinant attenuated vaccines. In this study, we constructed two CyHV-2 recombinant mutants, namely CyHV-2-Δ55-CP and CyHV-2-Δ57-CP, and evaluated their morphology, replication capability, pathogenicity, and ability to express heterologous proteins.

In the Alloherpesviridae family, several gene-deleted strains have been reported; however, current research mainly focuses on CCV and CyHV-3. In 1995, Zhang et al. reported a TK-deleted recombinant CCV strain that exhibited unaltered replicative capacity in channel catfish ovary (CCO) cells but showed significantly reduced virulence while inducing immune protection against CCV [28]. The infection kinetics of this TK-deleted CCV were similar to those of the wild-type CCV; however, the infection duration was shorter and shedding ability weaker [51]. Another study by Kunec et al. demonstrated that ORF12 gene deletion does affect in vitro replication of CCV [52], although its in vivo virulence was not determined. Vanderheijden et al., on the other hand, reported an attenuated CCV strain V60 with a large deletion observed in ORF50 gene (which may encode a secreted glycoprotein) [53], although it was not conclusively proven whether attenuation of virulence directly resulted from ORF50 segment deletion.

In CyHV-3, the TK gene initially garnered attention. Costes et al. generated ORF16 (putative G protein-coupled receptor)-deleted and ORF55 (TK)-excised CyHV-3 mutants; however, only the ORF55-excised mutant exhibited partially attenuated virulence [54]. Fuchs et al. constructed a series of CyHV-3 recombinant mutants with deletions in ORF55, ORF123 (deoxyuridine triphosphate pyrophosphatase), ORF141 (large subunit of ribonucleotide reductase), and both ORF55 and ORF123 genes [29]. The results indicated that all mutants can replicate in vitro, with only the ORF141-deleted CyHV-3 mutant showing reduced replication capability in vitro, while single-gene deletions of either ORF55 or ORF123 resulted in partial attenuation of CyHV-3 virulence [29]. Subsequent studies have revealed that double deletions of both ORF55 and ORF123 genes significantly attenuate virulence and induce significant immune protection in CyHV-3, making them a potential candidate for a recombinant attenuated vaccine [30,55]. Vancsok et al. created gene deletion mutants for 16 predicted virion transmembrane proteins (VTPs) in CyHV-3, including ORFs 25, 32, 59, 64, 65, 81, 83, 99, 106, 108, 115, 131, 132, 136, 148, and 149 [56]. However, only the deletion of the ORF25 gene resulted in substantial attenuation but showed poor immune protection [57]. In another study, the extent of virulence attenuation in the double-gene deletion mutants of ORF148 and ORF149 remains insufficient to meet the requirements for recombinant attenuated vaccine candidates [57]. Although deleting members of the glycoprotein family may lead to virulence attenuation, their suitability as vaccine candidates is limited due to potential effects on immunogenicity and relatively high sensitivity to mutations in paralogous genes. A recent study has demonstrated that the deletion of the ORF150 gene lead to significant virulence attenuation and effective immune protection in CyHv-3 mutants [58]. However, this vaccine candidate still requires extensive field trials to assess its effectiveness in real aquaculture environments. As previously mentioned in the introduction, ORF57 has been identified as a dispensable key virulence gene in CyHV-3 and it is conserved within the CyHVs [25,27]. Furthermore, the safety and efficacy of ORF57-deleted CyHV-3 mutants as candidates for recombinant attenuated vaccines have also been validated [24,25,56].

In our study, there was no significant alterations in the morphology and in vitro replication capabilities of CyHV-2-Δ55-CP and CyHV-2-Δ57-CP, indicating that both ORF55 and ORF57 genes are dispensable in CyHV-2. In the in vivo virulence assay, partial attenuation of virulence was observed for both CyHV-2-Δ55-CP and CyHV-2-Δ57-CP, with a more pronounced reduction seen for CyHV-2-Δ57-CP. Although the function of orthologues of pORF57 in genus *Cyvirus* remains unknown, previous research has demonstrated their abundant presence in viral particles for both orthologues in CyHV-3 [59] and AngHV-1 [60], while pORF57 has been identified as a major immunogenic protein in CyHV-2 [61]. The deletion of the TK gene primarily affects viral replication in non-replicating cells lacking cellular TK [26]. However, this negative impact on TK-deleted viruses can be partially offset by a higher proportion of dividing cells found in juvenile fish [26]. This may contribute to the incomplete virulence attenuation of TK-deleted viruses. It should be noted that the potential impact of NNV-CP fusion protein on the replication capabilities of CyHV-2 remains unknown, but research has shown that the precursor protein α of RGNNV-CP can induce apoptosis in host cells [62]. Overall, although neither CyHV-2 mutants CyHV-2-Δ55-CP and CyHV-2-Δ57-CP achieved sufficient levels of virulence attenuation to meet the requirements for recombinant attenuated vaccine candidates, ORF55 and ORF57 genes still serve as reference targets for constructing multi-gene-deleted CyHV-2 mutants.

Furthermore, capsid protein (CP) is known as the sole structural protein of NNV and has been extensively studied for vaccine development through recombinant expression using various vectors and systems, such as bacteria [63], yeast [64], insect cells [65], avian cells [66], and plant cells [67]. In our study using CyHV-2 as viral vector, successful expression of the NNV-CP fusion protein was achieved in GiCF cells. Some NNV-CP fusion proteins can self-assemble into VLPs, with foreign proteins fused at their C-terminal displayed on their surface [68]. However, in this study, the assembly of NNV VLPs within GiCF cells was difficult due to strong interference caused by C-terminus fusion with large protein [68]. In some studies, NNV-CP fusion proteins without a VLP formation have also demonstrated strong immunogenicity and provided effective protection for grouper [63,69]. Groupers that received intraperitoneal injections of GiCF cell lysates containing live or inactivated CyHV-2-Δ57-CP also produced specific anti-NNV IgM antibodies. However, further research is needed to determine its capacity for inducing effective immune protection and the ability of CyHV-2 recombinant mutants to replicate in unnatural hosts. In summary, this study demonstrates that CyHV-2 has the ability to express foreign proteins, which elicit the production of specific IgM antibodies in vaccinated fish, suggesting its potential as a viral vector. This provides insights into the development of replicative vaccines expressing foreign proteins within hosts and expression systems for foreign proteins in sensitive cell lines. For instance, by replacing the key virulence gene with the glycoprotein gene of SVCV (spring viremia of carp virus, *Sprivivirus cyprinus*), it would be possible to construct bivalent recombinant attenuated vaccine candidates against the two main viruses affecting crucian carp: CyHV-2 and SVCV.

## 5. Conclusions

In conclusion, we successfully generated two CyHV-2 recombinant mutants, namely CyHV-2-Δ55-CP and CyHV-2-Δ57-CP, by inserting the NNV-CP fusion protein expression cassette while deleting the ORF55 or ORF57 gene. Although dispensable for viral replication in vitro, both ORF55 and ORF57 genes of CyHV-2 contribute to virulence in vivo. Additionally, our findings demonstrate that CyHV-2 can effectively express foreign proteins capable of inducing an antibody response in vaccinated fish. These preliminary research results highlight the potential of fully attenuated CyHV-2 as a viral vector for developing subunit vaccines or multivalent recombinant attenuated vaccines.

## Figures and Tables

**Table 1:** Primers used for constructing recombinant plasmids in this study.

Function	Name	Sequence (5′-3′)
For amplification of the NNV-CP gene segment	CP-F	ATGGTACGCAAAGGTGAGAAGAAATTG
	CP-R	GTTTTCCGAGTCAACCCTAGTGC
For construction of the NNV-CP gene segment with overlapping sequence termini (lowercase)	CP-*Eco*RI-F	gatctcgagctcaagcttcgaattcATGGTACGCAAAGGTGAGAAGAAATTG
	CP-Overlap-R	gtgggcttgtactcggtcatGTTTTCCGAGTCAACCCTAGTGC
For amplification of the Puro^r^ gene segment	Puro-F	ATGACCGAGTACAAGCCCACG
	Puro-R	GGCACCGGGCTTGCG
For construction of the Puro^r^ gene segment with overlapping sequence termini (lowercase)	Puro-Overlap-F	ctagggttgactcggaaaacATGACCGAGTACAAGCCCACG
	Puro-*Bam*HI-R	tgctcaccatggtggcgatggatctGGCACCGGGCTTGCG
For amplification of the NNV-CP fusion protein expression cassette	CP-box-F	CGTTACATAACTTACGGTAAATGGCCC
	CP-box-R	TTACTTGTACAGCTCGTCCATGCC
For construction of the NNV-CP fusion protein expression cassette with overlapping sequence termini (lowercase)	Δ55-CP-*Kpn*I-F	ctgacaatcgttacacggacggtacCGTTACATAACTTACGGTAAATGGCCC
	Δ55-CP-*Eco*RI-R	ctctgagggttcgggagtgaagaattTTACTTGTACAGCTCGTCCATGCC
	Δ57-CP-*Bam*HI-F	tgacatcatgagcgggggatccCGTTACATAACTTACGGTAAATGGCCC
	Δ57-CP-*Eco*RI-R	ctttgggtttagcgccgaattcTTACTTGTACAGCTCGTCCATGCC
For amplification of the ORF55 upstream arm	Δ55-U-F	GGGTATGTTATCCTTGTTGATGGCG
	Δ55-U-R	GTCCGTGTAACGATTGTCAGCAG
For construction of the ORF55 upstream arm with overlapping sequence termini (lowercase)	Δ55-U-*Bam*HI-F	gcctgcaggtcgactctagaggatcGGGTATGTTATCCTTGTTGATGGCG
	Δ55-U-Overlap-R	cgggagtgaagaattcgacatctatggtaccGTCCGTGTAACGATTGTCAGCAG
For amplification of the ORF55 downstream arm (lowercase)	Δ55-D-F	TTCACTCCCGAACCCTCAGAGG
	Δ55-D-R	CGACTGGTTCATATCCAACAGAGAAGT
For construction of the ORF55 downstream arm with overlapping sequence termini (lowercase)	Δ55-D-Overlap-F	ttacacggacggtaccatagatgtcgaattcTTCACTCCCGAACCCTCAGAGG
	Δ55-D-*Eco*RI-R	aacagctatgaccatgattacgaattgCGACTGGTTCATATCCAACAGAGAAGT
For amplification of the ORF57 upstream arm	Δ57-U--F	AGCTTGTTTCTGAAACCAGAGATGC
	Δ57-U-R	CCCGCTCATGATGTCACACTTG
For construction of the ORF57 upstream arm with overlapping sequence termini (lowercase)	Δ57-U-*Bam*HI-F	gcctgcaggtcgactctagaggatcAGCTTGTTTCTGAAACCAGAGATGC
	Δ57-U-Overlap-R	tgggtttagcgccgaattcgacatctatggatccCCCGCTCATGATGTCACACTTG
For amplification of the ORF57 downstream arm	Δ57-D-F	GGCGCTAAACCCAAAGCTC
	Δ57-D-R	AGCAAGCTGCGCTCTGG
For construction of the ORF57 downstream arm with overlapping sequence termini (lowercase)	Δ57-D-Overlap-F	atcatgagcgggggatccatagatgtcgaattcGGCGCTAAACCCAAAGCTC
	Δ57-D-*Eco*RI-R	acagctatgaccatgattacgaattAGCAAGCTGCGCTCTGG

**Table 2:** Survival rates of common cultured crucian carp varieties challenged by CyHV-2-WT.

Varieties	Weight (g)	Survival Rates (%)		
		Intraperitoneal Injection	Immersion for 2 h	Immersion for 2 Days
Gibel carp var. CAS V (*C. auratus gibelio*)	8 ± 2	100	100	100
	200 ± 20	100	100	100
Gibel carp var. CAS III (*C. auratus gibelio*)	200 ± 20	100	100	100
Fang Zheng crucian carp (*C. auratus gibelio*)	200 ± 20	100	100	100
White crucian carps (*C. auratus cuvieri*)	200 ± 20	100	100	100
Goldfish (*C. auratus* L.)	8 ± 2	90	100	30
	100 ± 10	100	100	100

**Table 3:** Mean survival rates of goldfish challenged by recombinant or wild-type CyHV-2 strains.

Virus Concentration	Mean Survival Rates (%)		
	CyHV-2-WT	CyHV-2-Δ55-CP	CyHV-2-Δ57-CP
10^2^ TCID_50_/mL	89.17	93.33	92.5
10^3^ TCID_50_/mL	36.67	55	72.5
10^4^ TCID_50_/mL	29.17	37.5	47.5

## Data Availability

The data that support the findings of this study are available from the corresponding author upon reasonable request.
